# Large Anæurismal Tumor Successfully Treated by a New Method

**Published:** 1857-12

**Authors:** 


*Large Congenital Aneurismal Tumor successfully treated by a New Method.* By E. K. Sanborn, M. D., Lowell, Mass., Prof. of Surgery in the Berkshire Medical Institution.

The history of the following case is familiar to a large number of medical gentlemen, who as students and practitioners have witnessed the various expedients that have been resorted to, at various times during the last three years, at the clinique of the Berkshire Medical Institution, in attempts to arrest the growth of this tumor. As the disease is one of great surgical interest generally, I have thought the case worthy of record, and furnish the following brief account from minutes made at the time of the operation, with a drawing illustrating the successful method of treatment; and which, so far as I know, is new.

Mr. B., an intelligent young man, 20 years of age, was first brought before the class at the Medical College in Pittsfield, by his attending physician, Dr. Lawrence, of South Adams, Mass., in the autumn of 1855. He was somewhat pallid, and anæmic; but was in very good health, with the exception of a large, pulsating tumor, extending from near the ear to the top of the head, forward to the temple, and backward to the mastoid process of the temporal bone. It was not covered with hair; was of a bluish color, pulsated, *visibly*, over its whole extent, and communicated the peculiar anæurismal thrill to the ear. The rush of blood through the tumor caused a continued and loud ringing noise in the ear of the patient, and at night especially, this was often so disagreeable as to prevent sleep. Within a short time, there had also been several alarming attacks of hæmorrhage, of an arterial character, the blood coming from a small rupture of the thin walls of the tumor. These attacks usually occurred in the night, while the patient was asleep, and awoke him by causing throbbing in the tumor, and faintness. The bleeding had been arrested by long continued pressure.

Dr. Lawrence stated "that the tumor had first attracted notice soon after birth, as a small purple *nævus* in the scalp, just above the ear—that it had steadily increased with the growth of the patient, until the present time. The period when pulsation was first noticed could not be ascertained. The swelling had never been treated except by pressure—which had been thoroughly, but ineffectually tried, early in the history of the disease." Dr. T. Childs, then Professor of Surgery in the Berkshire Medical Institution, proposed to treat the disease by cutting off the arterial supply of blood. Accordingly, a large number of arteries leading to the tumor were tied. The *occipital*, *temporal* and several other arteries of the scalp, which had become greatly enlarged, were successively ligatured, but without any permanent benefit. The treatment by puncture with red-hot needles was also attempted, but was followed by profuse hæmorrhage, and it was abandoned. Finally, at the last clinique of the term of 1855, Prof. Childs tied the common carotid of the side corresponding to the tumor. The size of the tumor was sensibly diminished by this operation, and it was thought that, conjoined with local pressure, it might effect a cure. But pulsation was reëstablished soon after the separation of the ligature, and the tumor again began to increase.

During the autumn session of 1856, Mr. B. was again brought before the class, having recently suffered severely from hæmorrhage, and showing the effects of loss of blood in his countenance. The case now came under my care; and as the young man himself was quite desirous of having the carotid of the other side ligatured, a day was appointed for the operation. On reviewing the history of previous operations, and examining closely the nature of this tumor, in reference to further procedure, I was led to make a change in the plan of treatment, by the following considerations:

1st. That the tumor was composed of arteries and veins—the bulk of the tumor consisting of dilated veins; and therefore any operation performed on the supplying arteries would not materially change the structure of the tumor, and would be unavailing.

2d. Ligature of the carotid had shown that the tumor would retain a marked size, and pulsation, with a very small supply of blood; and as a year had elapsed since the operation, tying the other carotid would not be likely to entirely stop the passage of blood through the tumor, on account of the collateral circulation.

Accordingly, the idea of this operation was abandoned, and the subsequent treatment was directed to the tumor itself. The operation at first performed, was similar to that of tying a varicose vein over a pin or needle. A long needle was entered through the healthy scalp, opposite the middle of the tumor and about an inch from its border, and carried under the tumor, following the skull closely, and coming out at a corresponding point on the opposite side; great care being taken not to penetrate the cavity of the tumor itself. A strong silk ligature was then twisted over the ends of the needle, and drawn as tightly as possible. This operation was repeated five or six times in different parts of the tumor. The needles were removed as soon as they were loosened by ulceration, which was usually in about a week. Under this treatment, the tumor perceptibly diminished in size, and, at times, the pulsations ceased; but it failed to arrest the circulation through the tumor, for the reason that as the ligature ulcerated through the folds of scalp which were necessarily gathered up between the needle and the silk, the pressure was diminished, the circulation was reëstablished, and at the end of three months it was found that, although smaller, the tumor was not radically changed in character.

The difficulty above alluded to was finally obviated by the plan indicated in the wood cut.* A long needle was thrust under the tumor as before; then, instead of the twisted suture, another needle, corresponding in length to the first, was placed over it, on the outside of the tumor, and the two ends were then tied together, so that the opposite walls of the tumor were brought in contact with each other by a sort of clamp. The portion of the tumor and scalp embraced between the needles thus arranged, sloughed, and the lower needle ulcerated entirely through, and was lifted out or through the tumor, on the sixth day, without hæmorrhage.

Three others were then put in, and, at one time, the patient had four of these needles, fastened in the way described, and embracing the tumor in various directions, without suffering serious inconvenience. In performing this operation, the two needles were made fast together at one end, while the other ends were brought together gradually, the force being applied somewhat after the fashion of that of the ordinary nut-cracker. The pulsationed disappeared after the application of the second needle: and I recently had the pleasure of presenting my patient to the class, entirely cured—the cicatrices left by the needles alone showing the situation of the former tumor. —*Boston Med. & Surg. Journal.*

---

*Excito-Secretory System—New Claimants of the Discovery.*—Our readers are aware that after the application of the doctrine of reflex nervous action to the process of secretion, by Dr. Marshall Hall, the priority of the suggestion was claimed by Dr. Campbell, of Augusta, Ga., and subsequently acknowledged by the illustrious English physiologist. Of late, Dr. Campbell's claim has been contested by two other American physicians. In the

* The wood cut which accompanies the article in the Boston Jour. is omitted. F.

September number of the Medical Independent, Dr. J. Adams Allen, Professor of Materia Medica in the University of Michigan, announced that the excito-secretory doctrine had been publicly taught and illustrated by him, while professor at the Indiana Medical College, as early as November, 1848, that is, two years before the date claimed by Dr. Campbell for *his* announcement. But in the November number of the same Journal, we find an article by Dr. Martyn Paine, of New York, who states that the idea of the excito-secretory function has been publicly taught by him ever since the year 1841, in his lectures at the University of New York, and is embodied in his "Institutes of Medicine," published in 1847; and that, moreover, the idea of the *generalization* of excito-motory action throughout the system, which Dr. Marshall Hall believed originated with himself, may be found in the same work.—*Ibid.*

